# Differential genome-wide gene expression profiling of bovine largest and second-largest follicles: identification of genes associated with growth of dominant follicles

**DOI:** 10.1186/1477-7827-8-11

**Published:** 2010-02-05

**Authors:** Ken-Go Hayashi, Koichi Ushizawa, Misa Hosoe, Toru Takahashi

**Affiliations:** 1 Reproductive Biology Research Unit, Division of Animal Science, National Institute of Agrobiological Sciences, Tsukuba 305-8602, Japan

## Abstract

**Background:**

Bovine follicular development is regulated by numerous molecular mechanisms and biological pathways. In this study, we tried to identify differentially expressed genes between largest (F1) and second-largest follicles (F2), and classify them by global gene expression profiling using a combination of microarray and quantitative real-time PCR (QPCR) analysis. The follicular status of F1 and F2 were further evaluated in terms of healthy and atretic conditions by investigating mRNA localization of identified genes.

**Methods:**

Global gene expression profiles of F1 (10.7 +/- 0.7 mm) and F2 (7.8 +/- 0.2 mm) were analyzed by hierarchical cluster analysis and expression profiles of 16 representative genes were confirmed by QPCR analysis. In addition, localization of six identified transcripts was investigated in healthy and atretic follicles using in situ hybridization. The healthy or atretic condition of examined follicles was classified by progesterone and estradiol concentrations in follicular fluid.

**Results:**

Hierarchical cluster analysis of microarray data classified the follicles into two clusters. Cluster A was composed of only F2 and was characterized by high expression of 31 genes including IGFBP5, whereas cluster B contained only F1 and predominantly expressed 45 genes including CYP19 and FSHR. QPCR analysis confirmed AMH, CYP19, FSHR, GPX3, PlGF, PLA2G1B, SCD and TRB2 were greater in F1 than F2, while CCL2, GADD45A, IGFBP5, PLAUR, SELP, SPP1, TIMP1 and TSP2 were greater in F2 than in F1. In situ hybridization showed that AMH and CYP19 were detected in granulosa cells (GC) of healthy as well as atretic follicles. PlGF was localized in GC and in the theca layer (TL) of healthy follicles. IGFBP5 was detected in both GC and TL of atretic follicles. GADD45A and TSP2 were localized in both GC and TL of atretic follicles, whereas healthy follicles expressed them only in GC.

**Conclusion:**

We demonstrated that global gene expression profiling of F1 and F2 clearly reflected a difference in their follicular status. Expression of stage-specific genes in follicles may be closely associated with their growth or atresia. Several genes identified in this study will provide intriguing candidates for the determination of follicular growth.

## Background

The final stage of bovine follicular development occurs in a wave-like fashion [1,2]. During a wave, increase of follicle-stimulating hormone (FSH) induces recruitment of a cohort of follicles beyond 4 mm in diameter and usually a single follicle is selected as a dominant follicle (DF) [3,4]. Although the DF continues to grow by transition of gonadotropin dependency from FSH to luteinizing hormone (LH) and secretes large quantities of estradiol (E_2_), the remaining subordinate follicles (SFs) cease to grow, then undergo atresia [5]. It is well documented that increased expression of LH receptor (LHR) in granulosa cells (GC) and specific changes of intrafollicular factors such as the insulin-like growth factor (IGF) and inhibin-activin-follistatin systems play a critical role in E_2 _production in the DF [6,7]. Therefore, regulatory mechanisms of follicular development are closely associated with complex interactions between follicular local paracrine/autocrine factors and endocrine hormones.

Increasing evidence using global gene expression analysis such as a DNA microarray, suppression subtractive hybridization and serial analysis of gene expression have identified numerous genes in various aspects of bovine follicular development [8-18]. Some studies compared the gene expression profiles between DF and SF around the time of follicular selection. They showed that DF up-regulates genes regulating E_2 _synthesis, anti-apoptosis, cell proliferation and gene transcription. Conversely, SF enhanced the expression of genes associated with pro-apoptosis and cell death compared with the DF [8,9,13,14]. Recent studies found that 93 mostly novel genes were differently expressed in the GC of newly selected DF compared with SF and/or growing cohort follicles whereas most of these genes were down-regulated in the GC of preovulatory follicles during final maturation before the LH surge [15,17]. Growth of a DF during 2-5.5 days following follicular wave emergence was associated with a decrease in genes encoding proliferation and pro-apoptotic factors and an increase in genes regulating anti-apoptotic factors [12]. An increase in follicular diameter during follicular growth was accompanied by alteration of gene expression regulating some growth factors and cytokines [16,18]. Ndiaye *et al*. identified a subset of novel genes down-regulated in preovulatory follicles after human chorionic gonadotropin (hCG) stimulation compared with DF, which may contribute to ovulation and luteinization [11].

These previous studies lead us to suggest that gene expression profiles in individual follicles reflect their developmental status, thus each follicle can be classified by differences in gene expression profiles. On the other hand, details of the genetic processes and biological pathways regulating bovine follicular development still remain to be elucidated. We consider that investigating the global gene expression of follicles after selection can help to understand the molecular mechanisms responsible for the regulation and control of follicular development and atresia. Therefore, in this study, we tried to classify the largest (F1) and second-largest (F2) follicles according to differences in gene expression profiles and to identify differentially expressed genes between the groups using a combination of microarray analysis and quantitative real-time PCR (QPCR) analysis. In addition, spatial expression profiles of several identified genes were investigated using *in situ *hybridization in healthy and atretic follicles classified based on follicular fluid (FF) concentration of steroids.

## Methods

### Experiment 1: classification of F1 and F2 and identification of genes by microarray analysis and QPCR analysis

#### Sample collection and RNA extraction

Paired ovaries were obtained from four pregnant Japanese Black cows in the institute ranch less than 10 min after slaughtering. These cows were pregnant and slaughtered for another study. Both F1 and F2 were dissected from the ovaries. Then, the surrounding stroma and theca externa were removed from the follicular walls. We collected three F1 and three F2 from four cows because two cows had both F1 and F2 collected whereas one cow had only a F1 collected and another cow had only a F2 collected. The follicles were snap-frozen and stored at -80°C until RNA extraction. Total RNA from the follicular wall (i.e., granulosa plus theca interna) was extracted from each follicle using ISOGEN (NipponGene, Tokyo, Japan) according to the manufacturer's instructions. All procedures for animal experiments were carried out in accordance with guidelines approved by the Animal Ethics Committee of the National Institute of Agrobiological Sciences for the use of animals.

#### Microarray analysis

A custom-made bovine oligonucleotide microarray fabricated by Agilent Technologies (Santa Clara, CA, USA) was used in this study. Sixty-mer nucleotide probes for customized microarray were synthesized on a glass slide. The annotated bovine oligonucleotide array represented 10263 sequences 4466 of which were known bovine genes, 5697 were unknown sequences and possible candidates for novel bovine genes, and 100 internal references.

We performed one-color microarray using five follicles (three F1 and two F2). Fluorescence-labeled (Cy3) cRNA probes were prepared from 150-300 ng of total RNA of each follicle using a Low RNA Input Linear Amplification Kit (Agilent Technologies). Labeled cRNA probes (750 ng each) were hybridized to the customized microarray in hybridization buffer (Gene Expression Hybridization Kit, Agilent Technologies) at 60°C for 17 h. After hybridization, the arrays were washed with 6 × SSC, 0.005% Triton X-102 at room temperature for 10 min, followed by 5-min washes in 0.1 × SSC, 0.005% Triton X-102 at 4°C. Hybridized arrays were blow dried with N_2 _gas and scanned using an Agilent Microarray Scanner (Agilent Technologies), and Feature Extraction ver. 9.1 (Agilent Technologies) was used for image analysis and data extraction. Gene expression datasets were normalized using the median of the signal intensity for 100 *GAPDH *genes on a microarray platform as internal control.

After normalization, 3308 genes were left to use for further analysis. The relative abundance of individual genes between follicles was calculated by dividing the normalized value of the genes between each follicle. We used the normalized microarray data of genes that showed an expression level of more than 20-fold between at least two follicles for subsequent hierarchical cluster analysis. The data were transformed log_2 _values and hierarchical cluster analysis was performed using the TIGR MultiExperiment Viewer 4.0 (MeV 4.0) software program [19]. Two parameters (average linkage and cosine correlation) were selected for constructing the hierarchical tree. Compliance with Minimum Information About a Microarray Experiment (MIAME) [20] was assured by depositing all the data in the Gene Expression Omnibus (GEO) repository [21]. The GEO accession numbers are as follows. Platform: GPL9136; Samples: GSM453634, GSM453635, GSM453636, GSM453637 and GSM453638; Series: GSE18145.

#### Quantitative real-time RT-PCR analysis

To validate the results of microarray analysis, we confirmed mRNA expression of 16 representative genes using QPCR analysis. All six follicles were used in QPCR analysis. The procedures for QPCR were previously described [22]. Briefly, single-strand cDNA was reverse-transcribed from 50 ng of total RNA using MultiScribe™ reverse transcriptase with a random primer, dNTP mixture, MgCl_2 _and RNase inhibitor (Applied Biosystems, Foster City, CA, USA). The reverse transcription cycle consisted of 10 min annealing at 25°C, 30 min cDNA synthesis at 48°C and 5 min inactivation at 95°C. The primers were designed using the Primer Express computer software program (Applied Biosystems) based on the bovine sequences. The primer sequences for each gene are given in Table 1. Each QPCR reaction (25 μl) contained 1 μl cDNA template, 0.5 μl forward primer (20 μM), 0.5 μl reverse primer (20 μM), 12.5 μl Power SYBR^® ^Green PCR Master Mix (Applied Biosystems) and 10.5 μl nuclease-free water. The thermal cycling conditions included one cycle at 50°C for 2 min, one cycle at 95°C for 10 min, and 40 cycles at 95°C for 15 s and 60°C for 1 min. Each cDNA template was analyzed for quantitation in duplicate. QPCR and the resulting relative increase in reporter fluorescent dye emission were monitored in real time using an Mx3000P QPCR system (Stratagene, La Jolla, CA, USA). The relative difference in the initial amount of each mRNA species (or cDNA) was determined by comparing the cycle threshold values. To quantify the mRNA concentrations, standard curves for each gene were generated by serial dilution of the plasmid containing its cDNA. The melting curve for detecting the SYBR Green-based objective amplicon were confirmed because SYBR Green also detects double-stranded DNA including primer dimers, contaminating DNA and PCR products from misannealed primers. Contaminating DNA or primer dimers appear as a peak separate from the desired amplicon peak.

**Table 1:** Details of the primers used for quantitative real-time RT-PCR analysis

Gene name	GeneBank accession number	Primer	Sequences	Position
*AMH*	NM_173890	Forward	5'-ACACCGGCAAGCTCCTCAT-3'	1647-1665
		Reverse	5'-CACCATGTTTGGGACGTGG-3'	1714-1696
*CCL2*	NM_174006	Forward	5'-CGCTCAGCCAGATGCAATTA-3'	110-129
		Reverse	5'-GCCTCTGCATGGAGATCTTCTT-3'	186-165
*CYP19*	NM_174305	Forward	5'-TCCATGGGATTTTCCAGGC-3'	2050-2068
		Reverse	5'-TGGTGGCTTGTCTTTTCCAAC-3'	2123-2103
*FSHR*	NM_174061	Forward	5'-AATCTACCTGCTGCTCATAGCCTC-3'	1300-1323
		Reverse	5'-TTTGCCAGTCGATGGCATAG-3'	1376-1357
*GADD45A*	NM_001034247	Forward	5'-CCGCATTCATCACAGTGGAA-3'	592-611
		Reverse	5'-CATCACCGTTCAGGGAGATTAATC-3'	704-681
*GPX3*	NM_174077	Forward	5'-GCTTCCCCTGCAACCAATT-3'	357-375
		Reverse	5'-TCGAACATACTTGAGGGTGGCT-3'	433-412
*IGFBP5*	NM_001105327	Forward	5'-ACTGTGACCGCAAAGGGTTCT-3'	682-702
		Reverse	5'-TTCATCCCGTACTTGTCCACG-3'	778-758
*PlGF*	NM_173950	Forward	5'-TGAATGACTCACTCCCTCCATG-3'	877-898
		Reverse	5'-GGTCTGTCTTCTTTCTCTCACGTTC-3'	957-933
*PLAUR*	NM_174423	Forward	5'-CGCGGCCCTATGAATCAAT-3'	730-748
		Reverse	5'-CTGATGGTGTAGCTTGGGTTCC-3'	800-779
*PLA2G1B*	NM_174646	Forward	5'-GGCCTTCATCTGCAACTGTGA-3'	358-378
		Reverse	5'-TGTGCTCCTTGTTGTATGGCA-3'	428-408
*SCD*	NM_173959	Forward	5'-ATTCCCGACGTGGCTTTTTC-3'	659-678
		Reverse	5'-TTCTTTGACAGCTGGGTGTTTG-3'	729-708
*SELP*	NM_174183	Forward	5'-GTCAAGCAGGGCCACTGACTAT-3'	1700-1720
		Reverse	5'-TCACTAAGCCTGTTGTACCAGCTG-3'	203-2182
*SPP1*	NM_174187	Forward	5'-AGCCCTGAGCAAACAGACGAT-3'	304-324
		Reverse	5'-GCGTCGTCGGAGTCATTAGAGT-3'	380-359
*TIMP1*	NM_174471	Forward	5'-CTATGCTGCTGGTTGTGAGGAAT-3'	508-530
		Reverse	5'-TGAGTGTCGCTCTGCAGTTTG-3'	582-562
*TRB2*	NM_178317	Forward	5'-GACCTCAAGCTTCGGAAATTCA-3'	525-546
		Reverse	5'-CGTCATCTCCCCGCAGAATAT-3'	621-601
*TSP2*	NM_176872	Forward	5'-GGAAAACAAGTCATGGCGGA-3'	3845-3864
		Reverse	5'-TTGAGAGAAGACAAACAGACCCAG-3'	3928-3902
*GAPDH*	U85042	Forward	5'-ACCCAGAAGACTGTGGATGG-3'	444-463
		Reverse	5'-CAACAGACACGTTGGGAGTG-3'	621-602

### Experiment 2: localization of characteristic genes identified in experiment 1 in healthy and atretic follicles using in situ hybridization

#### Sample collection and storage

Ovaries containing follicles more than 8 mm in diameter were obtained from Japanese Black cows at local slaughterhouse. We used only follicles which have a transparent follicular wall and fluid and did not show any aspect of cystic follicles. Eleven follicles were collected and 200 μl of FF was aspirated from each follicle by a syringe fitted with a 27G needle. The FF was snap-frozen and stored at -30°C until hormone determinations. The follicles were dissected from the ovaries and fixed in 10% formalin, embedded in paraffin wax, and stored at 4°C until *in situ *hybridization.

#### Steroid hormone determinations

Concentrations of E_2 _and P_4 _in the FF samples were determined directly in duplicate using a time-resolved fluoroimmunoasssay (TR-FIA). The TR-FIA for E_2 _and P_4 _was performed as previously described by our laboratory [23,24]. The FF samples were diluted to 100-, 2000- and 5000-fold for E_2 _determination and 25-fold for P_4 _determination using charcoal-treated plasma (collected from adult Japanese-Black cows). Ranges of the standard curves were 5-200 pg/ml for E_2_ and 0.33-36 ng/ml for P_4_. The intra- and interassay coefficients of variation were 8.2 and 11.4% for E_2_, and 8.5 and 10.5% for P_4_, respectively.

#### In situ hybridization

We classified follicles into two groups based on relative levels of FF concentrations of E_2 _and P_4 _(E_2_/P_4 _≥ 1: healthy; E_2_/P_4 _< 1: atretic). Six representative genes differently expressed between F1 and F2 in experiment 1 were selected for *in situ *hybridyzation: anti-Mullerian hormone (*AMH*), cytochrome P450, family XIX (*CYP19*), growth arrest and DNA-damage-inducible, alpha (*GADD45A*), IGF binding protein 5 (*IGFBP5*), placental growth factor (*PlGF*) and thrombospondin 2 (*TSP2*). In these genes, *CYP19 *and *IGFBP5 *were selected as markers of healthy or atretic follicles since mRNA expression of *CYP19 *and *IGFBP5 *were up-regulated in the bovine DF and SF, respectively [25,26].

Digoxigenin (DIG)-labeled antisense and sense cRNA probes were prepared as previously described [27,28]. For hybridization, follicles were sectioned into 7 μm-thick sections. We performed *in situ *hybridization using an automated Ventana HX System Discovery with a RiboMapKit and a BlueMapKit (Roche Diagnostics, Basel, Switzerland) as previously described by our laboratory [27,28]. Briefly, the sections were hybridized with DIG-labeled probes in RiboHybe (Roche Diagnostics) hybridization solution at 65°C (*PlGF*) or 61°C (*AMH, CYP19, GADD45A, IGFBP5 *and *TSP2*) for 6 hours, then washed for 3 × 6 min in RiboWash (Roche Diagnostics) at 65°C and fixed in RiboFix (Roche Diagnostics) at 37°C, 10 min. The hybridization signals were detected with a rabbit polyclonal anti-digoxin antibody HRP conjugate (Dako Cytomation, Carpinteria, CA, USA) using an AmpMapKit (Roche Diagnostics). The hybridized slides were observed with a Leica DMRE HC microscope (Leica Microsystems, Wetzlar, Germany) and a Nikon Digital Sight DS-Fi1-L2 (Nikon, Tokyo, Japan).

#### Statistical analysis

In experiment 1, the expression ratio of each gene to *GAPDH *mRNA was calculated to adjust for variations in the QPCR reaction. The follicular diameter and the QPCR data in experiment 1 and concentrations of E_2 _and P_4 _and E_2_/P_4 _ratio in FF in experiment 2 were analyzed by Mann-Whitney's U test. Results were presented as the mean ± SEM. Statistical significance was considered to be at *P *< 0.05.

## Results

### Experiment 1: classification of F1 and F2 and identification of genes by microarray analysis and QPCR analysis

Mean diameter of F1 and F2 were 10.7 ± 0.7 and 7.8 ± 0.2 mm, respectively (*P *< 0.05).

#### Hierarchical cluster analysis of microarray data

**Figure 1:**
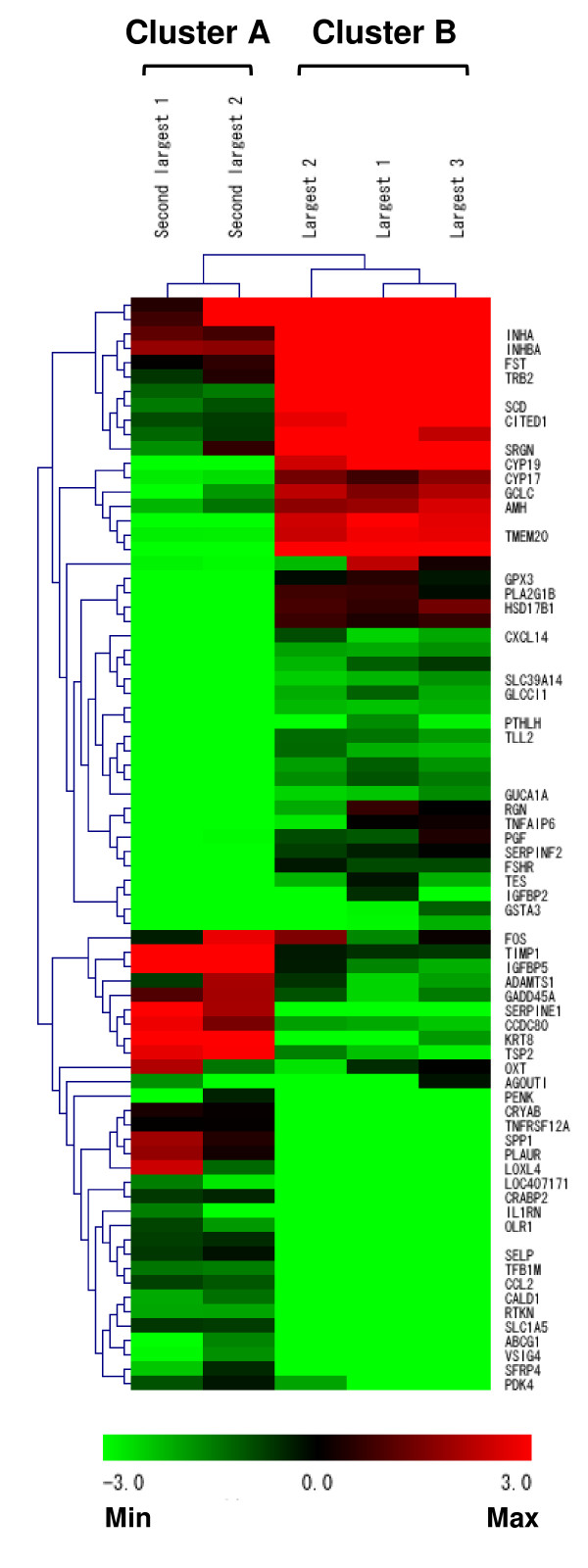
**Hierarchical cluster analysis of 76 differentially expressed genes in largest (F1) and second-largest follicles (F2)**. These genes were enhanced between at least two follicles by more than 20-fold. Red scale indicates relative higher expression level and green scale indicates relative lower expression level. The expression levels were transformed to log_2 _values. Dendrograms of sample axis (above matrix) and gene axis (to the left of matrix) represent overall similarities in gene expression profiles. Five follicles were classified into two major clusters (A and B). The follicles divided into cluster A were all F2 and the follicles divided into cluster B were all F1. The cluster A was characterized by highly expression of 31 genes, whereas the cluster B was predominately expressed 45 genes.

The expression level of 76 genes was enhanced between at least two follicles by more than 20-fold. Using the microarray data of these 76 genes, we performed a hierarchical cluster analysis and constructed a cluster heat map (Figure 1). As can be seen from the dendrogram of the sample axis, clustering analysis distinctly separated the five follicles into two clusters (A and B) based on their microarray expression profiles. Cluster A included two follicles that were both F2, whereas cluster B contained the remaining three follicles that were all F1. Cluster analysis also identified two major clusters in the gene axis. One cluster contained 31 genes that were relatively highly expressed in cluster A, while the other contained 45 genes that were relatively highly expressed in cluster B. The details of highly expressed genes in clusters A and B are listed in Table 2 and 3, respectively.

**Table 2:** List of differentially expressed genes in cluster A as compared with cluster B.

**Accession No**.	Gene symbol	Gene name
NM_001101080	ADAMTS1	ADAM metallopeptidase with thrombospondin type 1 motif, 1
NM_206843	AGOUTI	AGOUTI protein
NM_001098982	CCDC80	Coiled-coil domain containing 80
NM_174006	CCL2	Chemokine (C-C motif) ligand 2
NM_001008670	CRABP2	Cellular retinoic acid binding protein 2
NM_174290	CRYAB	Crystallin, alpha B
NM_001034247	GADD45A	Growth arrest and DNA-damage-inducible, alpha
NM_001077112	GSTA3	Glutathione S-transferase, alpha 3
NM_001105327	IGFBP5	Insulin-like growth factor binding protein 5
NM_174357	IL1RN	Interleukin 1 receptor antagonist
NM_001033610	KRT8	Keratin 8
NM_001001138	LOC407171	Fc gamma 2 receptor
NM_174384	LOXL4	Lysyl oxidase-like 4
NM_174132	OLR1	Oxidized low density lipoprotein (lectin-like) receptor 1
NM_176855	OXT	Oxytocin
NM_001101883	PDK4	Pyruvate dehydrogenase kinase, isozyme 4
NM_174141	PENK	Proenkephalin
NM_174423	PLAUR	Plasminogen activator, urokinase receptor
NM_001034681	RTKN	Rhotekin
NM_174183	SELP	Selectin P
NM_174137	SERPINE1	Serpin peptidase inhibitor, clade E (nexin, plasminogen activator inhibitor type 1), member 1
NM_001075764	SFRP4	Secreted frizzled-related protein 4
NM_174601	SLC1A5	Solute carrier family 1 (neutral amino acid transporter), member 5
NM_174187	SPP1	Secreted phosphoprotein 1
NM_003254	TIMP1	TIMP metallopeptidase inhibitor 1
NM_176872	TSP2	Thrombospondin 2
AW430112		Transcription factor B1, mitochondrial
BE721140		Transcribed locus
BP101259		Caldesmon, smooth muscle
XM_587930		Similar to ATP-binding cassette sub-family G member 1 (ABCG1), mRNA.
XM_869699		Similar to tumor necrosis factor receptor superfamily, member 12A

**Table 3:** List of differentially expressed genes in cluster B as compared with cluster A.

**Accession No**.	Gene symbol	Gene name
NM_173890	AMH	Anti-Mullerian hormone
NM_174518	CITED1	Cbp/p300-interacting transactivator, with Glu/Asp-rich carboxy-terminal domain, 1
NM_174304	CYP17	Cytochrome P450, subfamily XVII
NM_174305	CYP19	Cytochrome P450, family XIX, aromatase
NM_001034410	CXCL14	Chemokine (C-X-C motif) ligand 14
NM_182786	FOS	V-fos FBJ murine osteosarcoma viral oncogene homolog
NM_174061	FSHR	Follicle stimulating hormone receptor
NM_175801	FST	Follistatin
NM_001083674	GCLC	Glutamate-cysteine ligase, catalytic subunit
NM_174077	GPX3	Glutathione peroxidase 3
NM_174546	GUCA1A	guanylate cyclase activator 1A (retina)
NM_001102365	HSD17B1	Hydroxysteroid (17-beta) dehydrogenase 1
NM_174555	IGFBP2	Insulin-like growth factor binding protein 2, 36 kDa
NM_174094	INHA	Inhibin, alpha
NM_174363	INHBA	Inhibin, beta A (activin A, activin AB alpha polypeptide)
NM_173950	PlGF	Placental growth factor
NM_174646	PLA2G1B	Phospholipase A2, group IB (pancreas)
NM_174753	PTHLH	Parathyroid hormone-like hormone
NM_173957	RGN	Regucalcin (senescence marker protein-30)
NM_173959	SCD	Stearoyl-CoA desaturase (delta-9-desaturase)
NM_174670	SERPINF2	Serpin peptidase inhibitor, clade F (alpha-2 antiplasmin, pigment epithelium derived factor), member 2
NM_001098036	SLC39A14	Solute carrier family 39 (zinc transporter), member 14
NM_001025326	SRGN	Serglycin
NM_001076470	TMEM20	Transmembrane protein 20
NM_001007813	TNFAIP6	Tumor necrosis factor, alpha-induced protein 6
NM_178317	TRB2	TRB-2 protein
AW315959		13940 MARC 4BOV Bos taurus cDNA 5', mRNA sequence.
AW325368		16365 MARC 4BOV Bos taurus cDNA 5', mRNA sequence.
BE684800		186519 MARC 4BOV Bos taurus cDNA 5', mRNA sequence.
BI536463		393463 MARC 4BOV Bos taurus cDNA 5', mRNA sequence
BI536468		393469 MARC 4BOV Bos taurus cDNA 5', mRNA sequence.
BI537443		397313 MARC 4BOV Bos taurus cDNA 5', mRNA sequence.
BP102158		Transcribed locus
BP103904		BP103904 ORCS bovine liver cDNA Bos taurus cDNA clone ORCS25139 3', mRNA sequence.
BP104736		BP104736 ORCS bovine liver cDNA Bos taurus cDNA clone ORCS26135 3', mRNA sequence.
BP105513		BP105513 ORCS bovine liver cDNA Bos taurus cDNA clone ORCS27141 3', mRNA sequence.
BP107839		BP107839 ORCS bovine utero-placenta cDNA Bos taurus cDNA clone ORCS11248 3', mRNA sequence.
BP108716		Isolate UoG-BovSAGE-UK2 unknown mRNA
BP110155		Testis derived transcript (3 LIM domains)
BP110180		Transcribed locus
BP110819		BP110819 ORCS bovine utero-placenta cDNA Bos taurus cDNA clone ORCS11012 5', mRNA sequence.
BP111150		BP111150 ORCS bovine utero-placenta cDNA Bos taurus cDNA clone ORCS11443 5', mRNA sequence.
XM_614289		Similar to glucocorticoid induced transcript 1 (GLCCI1), mRNA.
XM_864694		Similar to tolloid-like 2, transcript variant 2 (TLL2), mRNA.

#### Quantitative PCR analysis of representative highly expressed genes in F1 and F2

**Figure 2:**
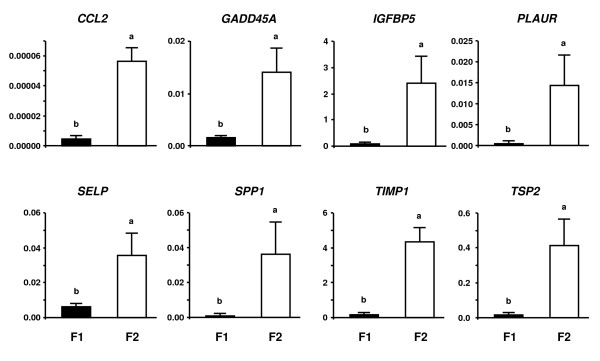
**QPCR analysis of representative eight genes (*CCL2, GADD45A, IGFBP5, PLAUR, SELP, SPP1, TIMP1 *and *TSP2*) in F1 and F2**. These genes were highly expressed in F2 (cluster A) compared with F1 (cluster B) in microarray analysis. The expression of mRNA was normalized to the expression of *GAPDH *measured in the same RNA preparation. The black bar and the white bar indicate the F1 and the F2, respectively. Data are shown as the mean ± SEM. Different letters denote significant differences (*P *< 0.05).

**Figure 3:**
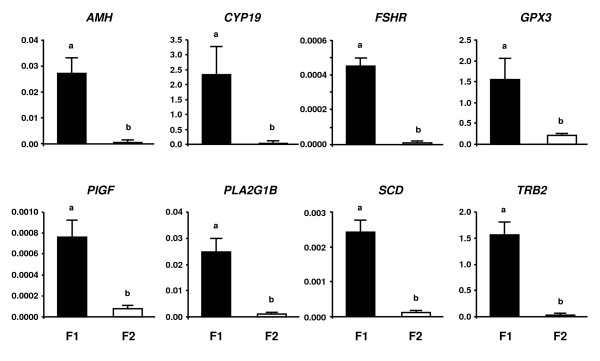
**QPCR analysis of representative eight genes (*AMH, CYP19, FSHR, GPX3, PlGF, PLA2G1B, SCD *and *TRB2*) in F1 and F2**. These genes were highly expressed in F1 (cluster B) compared with F2 (cluster A) in microarray analysis. The expression of mRNA was normalized to the expression of *GAPDH *measured in the same RNA preparation. The black bar and the white bar indicate the F1 and the F2, respectively. Data are shown as the mean ± SEM. Different letters denote significant differences (*P *< 0.05).

Figure 2 shows the results of QPCR analysis of the eight representative genes that were highly expressed in F2 (cluster A) compared with F1 (cluster B) in microarray analysis. Messenger RNA expression for chemokine ligand 2 (*CCL2*), *GADD45A*, *IGFBP5*, plasminogen activator urokinase receptor (*PLAUR*), secreted phosphoprotein 1 (*SPP1*), selectin P (*SELP*), tissue inhibitor of matrix metalloprotease-1 (*TIMP1*) and *TSP2 *was greater in the F2 than in the F1 (*P *< 0.05). The results of QPCR analysis of the eight representative genes that were highly expressed in the F1 as compared with the F2 in microarray analysis are shown in Figure 3. The expression of *AMH*, *CYP19*, *FSHR*, glutathione peroxidase 3 (*GPX3*), *PlGF*, phospholipase A2 group 1B (*PLA2G1B*), stearoyl-CoA desaturase (*SCD*) and tribbles homolog 2 (*TRB2*) mRNA was greater in the F1 than in the F2 (*P *< 0.05).

### Experiment 2: localization of characteristic genes identified in experiment 1 in healthy and atretic follicles using in situ hybridization

#### Follicular fluid concentrations of E_2 _and P_4 _in follicles

We classified follicles into healthy or atretic based on the relative concentrations of E_2 _and P_4 _in FF (healthy: E_2_/P_4 _ratio ≥1, atretic: E_2_/P_4 _ratio <1). From a total of 11 follicles, eight were categorized into healthy while the other three were atretic. Table 4 shows the characteristics of the follicles used in experiment 2. Healthy follicles had higher E_2 _and lower P_4 _concentrations in FF than atretic follicles. The E_2_/P_4 _ratio in FF was significantly higher in healthy follicles than in atretic follicles.

**Table 4:** Follicular fluid concentrations of estradiol (E_2_) and progesterone (P_4_) in examined follicles used in experiment 2.

Follicle	E_2 _(ng/ml)	P_4 _(ng/ml)	E_2_/P_4 _ratio
Healthy	180.0 ± 44.9	15.9 ± 15.3	4.3 ± 0.8
Atretic	41.4 ± 5.3*	387.7 ± 121.7*	0.1 ± 0.1*

Values are mean ± SEM

* *P *< 0.05 *vs*. healthy follicle

#### In situ hybridization of representative genes identified in experiment 1

**Figure 4:**
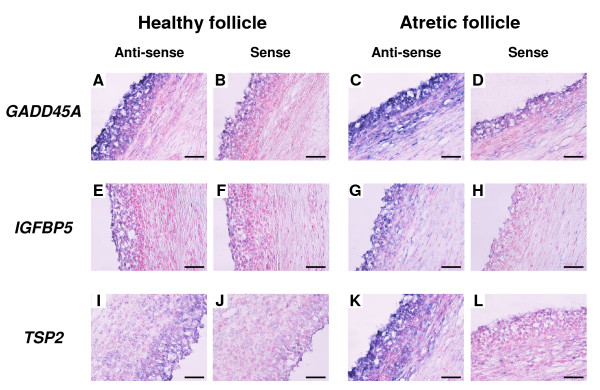
**Localization of *GADD45A, IGFBP5 *and *TSP2 *mRNA in healthy and atretic follicles**. These genes were expressed more in F2 than in F1 in QPCR analysis. (A, C, E, G, I and K) DIG-labeled anti-sense cRNA probes were used. (B, D, F, H, J and L) DIG-labeled sense cRNA probes were used. Seven-micrometer sections of bovine follicles were hybridized with each probe. *GADD45A *(A, B, C and D) and *TSP2 *(I, J, K and L) mRNA were found in both granulosa cells (GC) and theca layer (TL) of atretic follicle, whereas it was localized in only GC of healthy follicle. *IGFBP5 *mRNA (E, F, G and H) was localized in GC and TL of atretic follicle but not found in healthy follicle. Scale bar = 20 μm.

Figure 4 shows mRNA localization for *GADD45A*, *IGFBP5 *and *TSP2 *in healthy and atretic follicles by *in situ *hybridization. These genes were highly expressed in F2 than in F1 in microarray and QPCR analysis of experiment 1. *IGFBP5 *mRNA was localized in the GC and theca layer (TL) of atretic follicles but not in healthy follicles (Figure 4E, F, G and 4H). *GADD45A *(Figure 4A, B, C and 4D) and *TSP2 *(Figure 4I, J, K and 4L) mRNA were found in both GC and TL of atretic follicles but they were expressed in only GC of healthy follicles. No significant signals were detected with any sense probes (Figure 4B, D, F, H, J and 4L).

**Figure 5:**
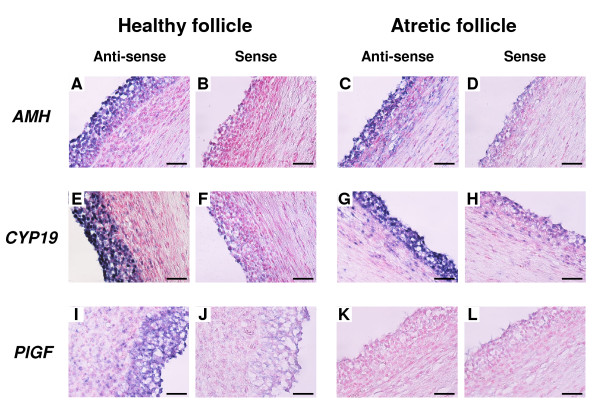
**Localization of *AMH, CYP19 *and *PlGF *mRNA in healthy and atretic follicles**. These genes were expressed more in F1 than in F2 in QPCR analysis. (A, C, E, G, I and K) DIG-labeled anti-sense cRNA probes were used. (B, D, F, H, J and L) DIG-labeled sense cRNA probes were used. Seven-micrometer sections of bovine follicles were hybridized with each probe. *AMH *(A, B, C and D) and *CYP19 *(E, F, G and H) mRNA was localized in granulosa cells (GC) of healthy as well as atretic follicles. *PlGF *mRNA (I, J, K and L) was found in GC and theca layer of healthy follicle but not atretic follicle. Scale bar = 20 μm.

Localization of *AMH*, *CYP19 *and *PlGF *mRNA in healthy and atretic follicles are shown in Figure 5. These genes were expressed more in the F1 than in the F2 in experiment 1. *AMH *(Figure. 5A, B, C and 5D) and *CYP19 *(Figure. 5E, F, G and 5H) mRNA was localized in GC of healthy as well as atretic follicles. *PlGF *mRNA was found in GC and TL of only healthy follicles but not atretic follicles (Figure. 5I, J, K and 5L). No significant signals were detected with any sense probes (Figure. 5B, D, F, H, J and 5L).

## Discussion

In this study, as expected, hierarchical cluster analysis of the microarray data classified F1 and F2 according to differences in gene expression profiles. In each follicular group, characteristic genes determining their developmental status were expressed. The F1 showed greater expression of genes responsible for enhancement of follicular E_2_ production than the F2. These genes were gonadotropin receptor (*FSHR*), steroidogenic enzymes (*CYP17, CYP19 *and *HSD17B1*) and inhibin-activin-follistatin system (*INHA*, *INHBA *and *FST*). It is well demonstrated that mRNA expression for *FSHR*, *CYP17*, *CYP19*, *INHA *and *INHBA *increases with the progress of bovine follicular development and is greater in DF than SF [8,12,29]. On the other hand, the F2 had greater expression of *IGFBP5 *mRNA than the F1. *IGFBP5 *mRNA expression dramatically increased in bovine atretic follicles compared with the DF [26]. Intrafollicular levels of IGFBP proteolytic activity and *IGFBPs *gene expression are important for bioavailability of free IGF within the follicle and play a crucial role for determining follicular dominance and fate [7,30]. Therefore, we evaluated the F1 were selected DF and the F2 were unselected SF.

Our evaluation of follicular status was confirmed to investigate *CYP19 *and *IGFBP5 *mRNA localization in healthy and atretic follicles in experiment 2 using *in situ *hybridization. *CYP19 *mRNA was abundantly expressed in healthy follicles but it was also expressed in atretic follicles while *IGFBP5 *mRNA was detected only in atretic follicles. Both *CYP19 *and *IGFBP5 *mRNA is hormonally regulated in bovine follicular cells [31-34], in addition, in situ hybridization is not quantitative and not be as sensitive as QPCR. Thus, small amounts of *CYP19 *and *IGFBP5 *mRNA may be detected or regulated in atretic and healthy follicles, respectively. Since we did not perform sample collection at a specific phase of follicular wave in experiment 1, detailed growth profiles of the follicles we used were unclear. However, our results demonstrate that randomly collected follicles can be divided into several groups by similarities of gene expression profiles among the follicles and suggest that gene expression profiles of examined follicles are closely associated with their development status.

Confirmation of microarray data by QPCR analysis successfully identified a set of genes differentially expressed between the F1 and F2. In addition, possible involvement of these genes in follicular development and/or atresia was further demonstrated to investigate mRNA localization in healthy and atretic follicles. The F1 showed greater expression of genes involved in follicular growth and survivability (*AMH*, *PLA2G1B*, *SCD2 *and *TRB2*) than the F2. High expression of these genes may be closely associated with the establishment and maintenance of follicular dominance. Although the functional role of AMH in antral follicle development is poorly understood, recent studies showed that both intrafollicular AMH concentration and *AMH *mRNA expression were highest in small antral follicles and then decreased with follicular growth, suggesting the involvement of AMH in bovine follicular recruitment and/or selection [16,35,36]. A recent study showed a significant decrease of *AMH *mRNA expression in late atretic follicles compared with healthy follicles [36], which is consistent with our present result. Furthermore, in agreement with previous studies [16,37], our *in situ *hybridization study showed that *AMH *mRNA was localized in only GC. High expression and clear localization of *AMH *mRNA in the GC of healthy large follicles implies that this growth factor has a plausible effect on the development of DF after follicular selection as well as recruitment.

PLA2 enzymes including PLA2G1B hydrolyze fatty acids from the sn-2 position of phospholipids with concomitant formation of lysophospholipids, which serve as precursor for lipid mediators such as lysophosphatidic acid (LPA) [38,39]. Released LPA has diverse biological activities including cell proliferation and differentiation, suppression of apoptosis and cytoskeleton modulation in reproductive tissues [39]. Because Diouf *et al*. reported that *PLA2G1B *mRNA expression in the GC of bovine preovulatory follicle decreased after hCG injection [40], PLA2G1B may mainly contribute to generation of LPA during DF growth before the LH surge.

SCD is a rate-limiting enzyme that catalyzes the synthesis of monounsaturated fatty acids, mainly palmitic and oleic acid [41]. Consistent with our result, *SCD *mRNA expression in bovine follicles was found to be highest in GC of DF than in cohort follicles before selection or SF [11,17]. Expression of *SCD2 *is hormonally regulated during follicular development because both SCD2 mRNA and protein expression in rat large follicles were stimulated by gonadotropin and IGF-I treatment [42]. Increase of monounsaturated acids synthesize by SCD2 activation during DF growth may be required to maintain membrane fluidity [43] and a major lipid reserve of oocytes [44].

Members of the TRB family including TRB2 interact and modulate the activity of mitogen-activated protein kinase (MAPK) which regulates cell proliferation, differentiation, apoptosis and survival [45]. These MAPK cascade protein levels were greater in DF than in SF [46]. In addition, it has been reported that *TRB2 *mRNA was constantly expressed between bovine small follicles and DF [11]. These studies and our present result suggest the potential role of TRB2 in the regulation of MAPK cascades in the growing DF.

The F2 are characterized by high expression of the genes involved in immune reaction (*CCL2*, *SELP *and *SPP1*). In bovine follicles, expression of *CCL2 *and *SPP1 *mRNAs and SELP protein was up-regulated in association with follicular development and ovulation [16,18,47]. Our results raise the possibility that these immune-related genes may be involved in bovine follicular atresia as well as follicular development and ovulation. Both CCL2 and SELP mediate induction of leukocyte emigration into extravascular inflammatory sites [48]. Although SPP1, also known as osteopontin, has diverse physiological functions, one of its potent actions is recruitment and retention of macrophages and T cells to inflamed sites [49]. Since number of leukocytes, lymphocytes and activated macrophages are increased in atretic follicles [50], CCL2, SELP and SPP1 participate in the regulation of inflammatory processes during follicular atresia to attract white blood cells.

The F2 are also characterized by high expression of genes regulating tissue remodeling (*TIMP1 *and *PLAUR*). Both plasminogen activator (PA)-plasmin and matrix metalloproteinase (MMP) systems play a crucial role in the degradation and remodeling of extracellular matrix associated with follicular development, ovulation and atresia [51]. Urokinase PA (uPA) receptor is a specific cell surface receptor for uPA and its principal role is to localize pericellular plasmin activity to induce extracellular matrix degradation [52]. A previous study showed that there was no difference in mRNA expression levels of uPA between non-atretic and atretic bovine follicles, whereas atretic follicles had lower FF protein level and mRNA expression of a PA inhibitor and higher FF plasmin activity than non-atretic follicles [53]. Therefore, the follicular PA-plasmin system may be primarily regulated by changes in their receptors and inhibitors' expressions. TIMP-1 is an intrinsic inhibitor of MMPs and preferentially binds to MMP-9 [54]. A previous study demonstrated that MMP-9 proenzyme (proMMP-9) protein in FF was detected only in atretic follicles but not in healthy follicles in cattle [55]. Atretic follicles may balance MMPs and TIMP-1 in response to an increase in proMMP-9 to control extracellular matrix degradation by MMP-9.

In the present study, we identified differential expression of two anti-apoptosis factors (*GADD45A *and *GPX3*) between the groups. Expression of *GADD45A *mRNA was greater in the F2 than in the F1. GADD45A controls cell cycle arrest, apoptosis induction and DNA damage repair in response to DNA damaging agents and growth arrest signals of genotoxic stress [56]. Our result implies that the atretic follicles suffered more severe DNA damage than healthy follicles. Indeed, we found in experiment 2 that the atretic follicles expressed *GADD45A *mRNA in both GC and TL whereas the healthy follicles expressed it only in GC. This result supports our microarray result and suggests an increase in the requirement of GADD45A activity for progression of apoptotic cell death in GC and TC during follicular atresia. On the other hand, *GPX3 *mRNA was found to be more greatly expressed in the F1 than in the F2. Glutathione peroxidase protects cells against oxidative damage to catalyze the reduction of free hydrogen peroxide and other hydroperoxides [57]. High oxidative stress can trigger apoptosis of follicular cells and induce atresia [58]. In cultured swine GC, *GPX3 *mRNA expression was upregulated by FSH treatment [59]. Thus GPX3 could prevent cell apoptosis from oxidative stress during growth of the healthy follicles. It is likely that follicular oxidative stress-response enzymes are expressed in a stage-dependent manner since mRNA expression of other anti-oxidative stress enzymes in bovine GC was increased in atretic DF than in healthy DF [60].

Providing a sufficient blood supply is essential for follicular growth [61,62]. A morphological study has demonstrated that bovine healthy DF has a high density and well developed capillaries in TL whereas atretic follicles has sparse and poorly developed capillaries [63]. In the present study, two genes regulating angiogenesis, *PlGF *and *TSP2*, were differentially expressed between the groups. *PlGF *was expressed most in F1 than in F2 and localized in both GC and TL of healthy follicles but not detected in atretic follicles. PlGF is a member of the vascular endothelial growth factor family and stimulates the proliferation of endothelial cells and supports angiogenesis [64,65]. Therefore, PlGF may contribute to follicular thecal angiogenesis via paracrine/autocrine action in healthy follicles as well as other angiogenic factors. In contrast to PlGF, TSP-2, a member of the TSP family, acts as a potent inhibitor of angiogenesis and induces endothelial cell apoptosis [66]. In experiment 1, *TSP2 *mRNA expression was greater in the F2 than in the F1. *TSP2 *mRNA level in the bovine follicles decreased in accordance with an increase in follicular diameter [67]. The same authors also showed that TSP protein was localized in both GC and TC of small follicles but in only in the GC of large follicles [67]. We demonstrated in experiment 2 that *TSP2 *mRNA was localized in both GC and TL of atretic follicles while it was expressed in only GC of healthy follicles. Recent studies have demonstrated that mRNA and protein expression of TSP-1, another antiangiogenic TSP, is upregulated in primate GC during progression of follicular atresia [68] and *TSP1 *mRNA abundance is decreased by IGF-I treatment in cultured porcine GC [69]. Thus, we speculate that *TSP2 *mRNA expression is maintained at high levels in follicular cells of atretic follicles whereas it decreases in healthy follicles. Highly expressed *TSP2 *mRNA in the follicles could negatively influence their angiogenesis. It may cause an insufficient supply of substrates essential for follicular growth, thereby affecting follicular hormone production and cell proliferation, and, as a result, inducing atresia.

## Conclusion

Microarray and QPCR analysis enabled us to classify uncharacterized bovine follicles and to evaluate their representative follicular status according to differences in global gene expression profiles. Our present study demonstrates that the expression of stage-specific genes in F1 and F2 may be closely associated with follicular growth and atresia. Several genes identified in this study will provide information on the genomic actions of intriguing candidates for the determinant of bovine follicular development.

## Competing interests

The authors declare that they have no competing interests.

## Authors' contributions

KGH participated in the design of the study, collected the materials, carried out all experiments and drafted the manuscript. KU collected the materials, carried out the microarray experiments and analysis, and helped to carry out QPCR and *in situ *hybridization. MH was responsible for all animal care, collected the materials and carried out the microarray experiments. TT supervised the study, collected the materials and helped to draft the manuscript. All authors read and approved the final manuscript.

## References

[B1] Sirois J Fortune JE Ovarian follicular dynamics during the estrous cycle in heifers monitored by real-time ultrasonography Biol Reprod 1988 39 308 317 10.1095/biolreprod39.2.308 3052602

[B2] Ginther OJ Knopf L Kastelic JP Temporal associations among ovarian events in cattle during oestrous cycles with two and three follicular waves J Reprod Fertil 1989 87 223 230 2621698 10.1530/jrf.0.0870223

[B3] Sunderland SJ Crowe MA Boland MP Roche JF Ireland JJ Selection, dominance and atresia of follicles during the oestrous cycle of heifers J Reprod Fertil 1994 101 547 555 7966007 10.1530/jrf.0.1010547

[B4] Ginther OJ Kot K Kulick LJ Wiltbank MC Emergence and deviation of follicles during the development of follicular waves in cattle Theriogenology 1997 48 75 87 10.1016/S0093-691X(97)00192-1 16728109

[B5] Ginther OJ Wiltbank MC Fricke PM Gibbons JR Kot K Selection of the dominant follicle in cattle Biol Reprod 1996 55 1187 1194 10.1095/biolreprod55.6.1187 8949873

[B6] Mihm M Crowe MA Knight PG Austin EJ Follicle wave growth in cattle Reprod Domest Anim 2002 37 191 200 10.1046/j.1439-0531.2002.00371.x 12173983

[B7] Fortune JE Rivera GM Yang MY Follicular development: the role of the follicular microenvironment in selection of the dominant follicle Anim Reprod Sci 2004 82-83 109 126 10.1016/j.anireprosci.2004.04.031 15271447

[B8] Sisco B Hagemann LJ Shelling AN Pfeffer PL Isolation of genes differentially expressed in dominant and subordinate bovine follicles Endocrinology 2003 144 3904 3913 10.1210/en.2003-0485 12933664

[B9] Evans AC Ireland JL Winn ME Lonergan P Smith GW Coussens PM Ireland JJ Identification of genes involved in apoptosis and dominant follicle development during follicular waves in cattle Biol Reprod 2004 70 1475 1484 10.1095/biolreprod.103.025114 14736815

[B10] Fayad T Levesque V Sirois J Silversides DW Lussier JG Gene expression profiling of differentially expressed genes in granulosa cells of bovine dominant follicles using suppression subtractive hybridization Biol Reprod 2004 70 523 533 10.1095/biolreprod.103.021709 14568916

[B11] Ndiaye K Fayad T Silversides DW Sirois J Lussier JG Identification of downregulated messenger RNAs in bovine granulosa cells of dominant follicles following stimulation with human chorionic gonadotropin Biol Reprod 2005 73 324 333 10.1095/biolreprod.104.038026 15829623

[B12] Mihm M Baker PJ Ireland JL Smith GW Coussens PM Evans AC Ireland JJ Molecular evidence that growth of dominant follicles involves a reduction in follicle-stimulating hormone dependence and an increase in luteinizing hormone dependence in cattle Biol Reprod 2006 74 1051 1059 10.1095/biolreprod.105.045799 16481595

[B13] Zielak AE Forde N Park SD Doohan F Coussens PM Smith GW Ireland JJ Lonergan P Evans AC Identification of novel genes associated with dominant follicle development in cattle Reprod Fertil Dev 2007 19 967 975 10.1071/RD07102 18076829

[B14] Zielak AE Canty MJ Forde N Coussens PM Smith GW Lonergan P Ireland JJ Evans AC Differential expression of genes for transcription factors in theca and granulosa cells following selection of a dominant follicle in cattle Mol Reprod Dev 2008 75 904 914 10.1002/mrd.20819 17948250

[B15] Baker PJ Fleming LM Mossa F Lonergan P Evans AC Mihm M Decreased mRNA expression of dominance maker genes in preovulatory compared to newly selected dominant follicles in cattle [abstract] Biol Reprod 2008 531

[B16] Skinner MK Schmidt M Savenkova MI Sadler-Riggleman I Nilsson EE Regulation of granulosa and theca cell transcriptomes during ovarian antral follicle development Mol Reprod Dev 2008 75 1457 1472 10.1002/mrd.20883 18288646 PMC5749411

[B17] Mihm M Baker PJ Fleming LM Monteiro AM O'Shaughnessy PJ Differentiation of the bovine dominant follicle from the cohort upregulates mRNA expression for new tissue development genes Reproduction 2008 135 253 265 10.1530/REP-06-0193 18239053

[B18] Liu Z Youngquist RS Garverick HA Antoniou E Molecular mechanisms regulating bovine ovarian follicular selection Mol Reprod Dev 2009 76 351 366 10.1002/mrd.20967 18932212

[B19] TM4 http://www.tm4.org/mev.html

[B20] MIAME http://www.mged.org/Workgroups/MIAME/miame.html

[B21] GEO http://www.ncbi.nlm.nih.gov/projects/geo/

[B22] Ushizawa K Takahashi T Hosoe M Ishiwata H Kaneyama K Kizaki K Hashizume K Global gene expression analysis and regulation of the principal genes expressed in bovine placenta in relation to the transcription factor AP-2 family Reprod Biol Endocrinol 2007 5 17 10.1186/1477-7827-5-17 17462098 PMC1867817

[B23] Takahashi T Hamanaka S Ikeda S Kobayashi J Hashizume K A direct time-resolved fluorescent immunoassay (TR-FIA) for measuring plasma progesterone concentration in sika doe (*Cervus nippon centralis*) J Reprod Dev 2001 47 119 123 10.1262/jrd.47.119

[B24] Takahashi T Hamanaka S Imai K Hashizume K A direct time-resolved fluoroimmunoassay (TR-FIA) for measuring plasma estradiol-17beta concentrations in cattle J Vet Med Sci 2004 66 225 229 10.1292/jvms.66.225 15107548

[B25] Bao B Garverick HA Smith GW Smith MF Salfen BE Youngquist RS Changes in messenger ribonucleic acid encoding luteinizing hormone receptor, cytochrome P450-side chain cleavage, and aromatase are associated with recruitment and selection of bovine ovarian follicles Biol Reprod 1997 56 1158 1168 10.1095/biolreprod56.5.1158 9160714

[B26] Santiago CA Voge JL Aad PY Allen DT Stein DR Malayer JR Spicer LJ Pregnancy-associated plasma protein-A and insulin-like growth factor binding protein mRNAs in granulosa cells of dominant and subordinate follicles of preovulatory cattle Domest Anim Endocrinol 2005 28 46 63 10.1016/j.domaniend.2004.06.002 15620806

[B27] Ushizawa K Kaneyama K Takahashi T Tokunaga T Tsunoda Y Hashizume K Cloning and expression of a new member of prolactin-related protein in bovine placenta: bovine prolactin-related protein-VII Biochem Biophys Res Commun 2005 326 435 441 10.1016/j.bbrc.2004.11.045 15582596

[B28] Ushizawa K Takahashi T Kaneyama K Hosoe M Hashizume K Cloning of the bovine antiapoptotic regulator, BCL2-related protein A1, and its expression in trophoblastic binucleate cells of bovine placenta Biol Reprod 2006 74 344 351 10.1095/biolreprod.105.042655 16221993

[B29] Bao B Garverick HA Expression of steroidogenic enzyme and gonadotropin receptor genes in bovine follicles during ovarian follicular waves: a review J Anim Sci 1998 76 1903 1921 9690647 10.2527/1998.7671903x

[B30] Canty MJ Boland MP Evans AC Crowe MA Alterations in follicular IGFBP mRNA expression and follicular fluid IGFBP concentrations during the first follicle wave in beef heifers Anim Reprod Sci 2006 93 199 217 10.1016/j.anireprosci.2005.06.033 16159699

[B31] Silva JM Price CA Effect of follicle-stimulating hormone on steroid secretion and messenger ribonucleic acids encoding cytochromes P450 aromatase and cholesterol side-chain cleavage in bovine granulosa cells in vitro Biol Reprod 2000 62 186 191 10.1095/biolreprod62.1.186 10611084

[B32] Silva JM Price CA Insulin and IGF-I are necessary for FSH-induced cytochrome P450 aromatase but not cytochrome P450 side-chain cleavage gene expression in oestrogenic bovine granulosa cells in vitro J Endocrinol 2002 174 499 507 10.1677/joe.0.1740499 12208671

[B33] Voge JL Aad PY Santiago CA Goad DW Malayer JR Allen D Spicer LJ Effect of insulin-like growth factors (IGF), FSH, and leptin on IGF-binding-protein mRNA expression in bovine granulosa and theca cells: quantitative detection by real-time PCR Peptides 2004 25 2195 2203 10.1016/j.peptides.2004.07.008 15572210

[B34] Voge JL Santiago CA Aad PY Goad DW Malayer JR Spicer LJ Quantification of insulin-like growth factor binding protein mRNA using real-time PCR in bovine granulosa and theca cells: effect of estradiol, insulin, and gonadotropins Domest Anim Endocrinol 2004 26 241 258 10.1016/j.domaniend.2003.11.002 15036378

[B35] Monniaux D Clemente N Touze JL Belville C Rico C Bontoux M Picard JY Fabre S Intrafollicular steroids and anti-mullerian hormone during normal and cystic ovarian follicular development in the cow Biol Reprod 2008 79 387 396 10.1095/biolreprod.107.065847 18448844

[B36] Rico C Fabre S Medigue C Clemente N Clement F Bontoux M Touze JL Dupont M Briant E Remy B Beckers JF Monniaux D Anti-mullerian hormone is an endocrine marker of ovarian gonadotropin-responsive follicles and can help to predict superovulatory responses in the cow Biol Reprod 2009 80 50 59 10.1095/biolreprod.108.072157 18784351

[B37] Takahashi M Hayashi M Manganaro TF Donahoe PK The ontogeny of mullerian inhibiting substance in granulosa cells of the bovine ovarian follicle Biol Reprod 1986 35 447 453 10.1095/biolreprod35.2.447 3533170

[B38] Eder AM Sasagawa T Mao M Aoki J Mills GB Constitutive and lysophosphatidic acid (LPA)-induced LPA production: role of phospholipase D and phospholipase A2 Clin Cancer Res 2000 6 2482 2491 10873103

[B39] Budnik LT Mukhopadhyay AK Lysophosphatidic acid and its role in reproduction Biol Reprod 2002 66 859 865 10.1095/biolreprod66.4.859 11906902

[B40] Diouf MN Sayasith K Lefebvre R Silversides DW Sirois J Lussier JG Expression of phospholipase A2 group IVA (PLA2G4A) is upregulated by human chorionic gonadotropin in bovine granulosa cells of ovulatory follicles Biol Reprod 2006 74 1096 1103 10.1095/biolreprod.105.048579 16510840

[B41] Nakamura MT Nara TY Structure, function, and dietary regulation of delta6, delta5, and delta9 desaturases Annu Rev Nutr 2004 24 345 376 10.1146/annurev.nutr.24.121803.063211 15189125

[B42] Moreau C Froment P Tosca L Moreau V Dupont J Expression and regulation of the SCD2 desaturase in the rat ovary Biol Reprod 2006 74 75 87 10.1095/biolreprod.105.044545 16207839

[B43] Abel S Smuts CM de Villiers C Gelderblom WC Changes in essential fatty acid patterns associated with normal liver regeneration and the progression of hepatocyte nodules in rat hepatocarcinogenesis Carcinogenesis 2001 22 795 804 10.1093/carcin/22.5.795 11323400

[B44] McEvoy TG Coull GD Broadbent PJ Hutchinson JS Speake BK Fatty acid composition of lipids in immature cattle, pig and sheep oocytes with intact zona pellucida J Reprod Fertil 2000 118 163 170 10.1530/reprod/118.1.163 10793638

[B45] Kiss-Toth E Bagstaff SM Sung HY Jozsa V Dempsey C Caunt JC Oxley KM Wyllie DH Polgar T Harte M O'neill LA Qwarnstrom EE Dower SK Human tribbles, a protein family controlling mitogen-activated protein kinase cascades J Biol Chem 2004 279 42703 42708 10.1074/jbc.M407732200 15299019

[B46] Ryan KE Casey SM Canty MJ Crowe MA Martin F Evans AC Akt and Erk signal transduction pathways are early markers of differentiation in dominant and subordinate ovarian follicles in cattle Reproduction 2007 133 617 626 10.1530/REP-06-0130 17379656

[B47] Rohm F Spanel-Borowski K Eichler W Aust G Correlation between expression of selectins and migration of eosinophils into the bovine ovary during the periovulatory period Cell Tissue Res 2002 309 313 322 10.1007/s00441-002-0602-3 12172791

[B48] Vestweber D Blanks JE Mechanisms that regulate the function of the selectins and their ligands Physiol Rev 1999 79 181 213 9922371 10.1152/physrev.1999.79.1.181

[B49] Mazzali M Kipari T Ophascharoensuk V Wesson JA Johnson R Hughes J Osteopontin--a molecule for all seasons Qjm 2002 95 3 13 10.1093/qjmed/95.1.3 11834767

[B50] Suzuki T Sasano H Takaya R Fukaya T Yajima A Date F Nagura H Leukocytes in normal-cycling human ovaries: immunohistochemical distribution and characterization Hum Reprod 1998 13 2186 2191 10.1093/humrep/13.8.2186 9756294

[B51] Ny T Wahlberg P Brandstrom IJ Matrix remodeling in the ovary: regulation and functional role of the plasminogen activator and matrix metalloproteinase systems Mol Cell Endocrinol 2002 187 29 38 10.1016/S0303-7207(01)00711-0 11988309

[B52] Blasi F Carmeliet P uPAR: a versatile signalling orchestrator Nat Rev Mol Cell Biol 2002 3 932 943 10.1038/nrm977 12461559

[B53] Cao M Buratini J Jr Lussier JG Carriere PD Price CA Expression of protease nexin-1 and plasminogen activators during follicular growth and the periovulatory period in cattle Reproduction 2006 131 125 137 10.1530/rep.1.00849 16388016

[B54] Goldberg GI Strongin A Collier IE Genrich LT Marmer BL Interaction of 92-kDa type IV collagenase with the tissue inhibitor of metalloproteinases prevents dimerization, complex formation with interstitial collagenase, and activation of the proenzyme with stromelysin J Biol Chem 1992 267 4583 4591 1311314

[B55] Khandoker MA Imai K Takahashi T Hashizume K Role of gelatinase on follicular atresia in the bovine ovary Biol Reprod 2001 65 726 732 10.1095/biolreprod65.3.726 11514334

[B56] Zhan Q Gadd45a a p53- and BRCA1-regulated stress protein, in cellular response to DNA damage Mutat Res 2005 569 133 143 15603758 10.1016/j.mrfmmm.2004.06.055

[B57] Yu BP Cellular defenses against damage from reactive oxygen species Physiol Rev 1994 74 139 162 8295932 10.1152/physrev.1994.74.1.139

[B58] Tilly JL Tilly KI Inhibitors of oxidative stress mimic the ability of follicle-stimulating hormone to suppress apoptosis in cultured rat ovarian follicles Endocrinology 1995 136 242 252 10.1210/en.136.1.242 7828537

[B59] Bonnet A Frappart PO Dehais P Tosser-Klopp G Hatey F Identification of differential gene expression in in vitro FSH treated pig granulosa cells using suppression subtractive hybridization Reprod Biol Endocrinol 2006 4 35 10.1186/1477-7827-4-35 16827936 PMC1533831

[B60] Valdez KE Cuneo SP Turzillo AM Regulation of apoptosis in the atresia of dominant bovine follicles of the first follicular wave following ovulation Reproduction 2005 130 71 81 10.1530/rep.1.00430 15985633

[B61] Zeleznik AJ Schuler HM Reichert LE Jr Gonadotropin-binding sites in the rhesus monkey ovary: role of the vasculature in the selective distribution of human chorionic gonadotropin to the preovulatory follicle Endocrinology 1981 109 356 362 10.1210/endo-109-2-356 6265188

[B62] Acosta TJ Hayashi KG Matsui M Miyamoto A Changes in follicular vascularity during the first follicular wave in lactating cows J Reprod Dev 2005 51 273 280 10.1262/jrd.16092 15699584

[B63] Jiang JY Macchiarelli G Tsang BK Sato E Capillary angiogenesis and degeneration in bovine ovarian antral follicles Reproduction 2003 125 211 223 10.1530/rep.0.1250211 12578535

[B64] Maglione D Guerriero V Viglietto G Delli-Bovi P Persico MG Isolation of a human placenta cDNA coding for a protein related to the vascular permeability factor Proc Natl Acad Sci USA 1991 88 9267 9271 10.1073/pnas.88.20.9267 1924389 PMC52695

[B65] Carmeliet P Moons L Luttun A Vincenti V Compernolle V De Mol M Wu Y Bono F Devy L Beck H Scholz D Acker T DiPalma T Dewerchin M Noel A Stalmans I Barra A Blacher S Vandendriessche T Ponten A Eriksson U Plate KH Foidart JM Schaper W Charnock-Jones DS Hicklin DJ Herbert JM Collen D Persico MG Synergism between vascular endothelial growth factor and placental growth factor contributes to angiogenesis and plasma extravasation in pathological conditions Nat Med 2001 7 575 583 10.1038/87904 11329059

[B66] Streit M Riccardi L Velasco P Brown LF Hawighorst T Bornstein P Detmar M Thrombospondin-2: a potent endogenous inhibitor of tumor growth and angiogenesis Proc Natl Acad Sci USA 1999 96 14888 14893 10.1073/pnas.96.26.14888 10611308 PMC24743

[B67] Greenaway J Gentry PA Feige JJ LaMarre J Petrik JJ Thrombospondin and vascular endothelial growth factor are cyclically expressed in an inverse pattern during bovine ovarian follicle development Biol Reprod 2005 72 1071 1078 10.1095/biolreprod.104.031120 15616224

[B68] Thomas FH Wilson H Silvestri A Fraser HM Thrombospondin-1 expression is increased during follicular atresia in the primate ovary Endocrinology 2008 149 185 192 10.1210/en.2007-0835 17884943

[B69] Grado-Ahuir JA Aad PY Ranzenigo G Caloni F Cremonesi F Spicer LJ Microarray analysis of insulin-like growth factor-I-induced changes in messenger ribonucleic acid expression in cultured porcine granulosa cells: possible role of insulin-like growth factor-I in angiogenesis J Anim Sci 2009 87 1921 1933 10.2527/jas.2008-1222 19251926

